# Solvent-Assisted Crystallization of an α-Fe_2_O_3_ Electron Transport Layer for Efficient and Stable Perovskite Solar Cells Featuring Negligible Hysteresis

**DOI:** 10.1021/acsomega.3c01405

**Published:** 2023-05-09

**Authors:** Akbar Ali Qureshi, Sofia Javed, Muhammad Aftab Akram, Lukas Schmidt-Mende, Azhar Fakharuddin

**Affiliations:** † School of Chemical & Materials Engineering, National University of Sciences & Technology, Islamabad 44000, Pakistan; ‡ Department of Materials Science & Engineering, Pak-Austria Fachhochschule, Institute of Applied Sciences & Technology, Haripur 22650, Pakistan; § Department of Physics, University of Konstanz, Konstanz 78464, Germany

## Abstract

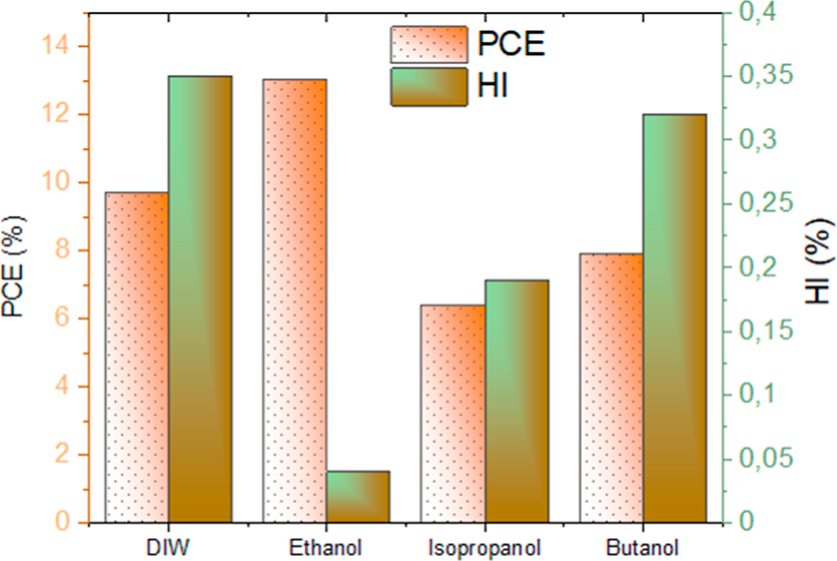

Inorganic–organic metal halide perovskite solar cells (PSCs) show power conversion efficiency values approaching those of state-of-the-art silicon solar cells. In a quest to find suitable charge transport materials in PSCs, hematite (α-Fe_2_O_3_) has emerged as a potential electron transport layer (ETL) in n–i–p planar PSCs due to its low cost, UV light stability, and nontoxicity. Yet, the performance of α-Fe_2_O_3_-based PSCs is far lower than that of state-of-the-art PSCs owing to the poor quality of the α-Fe_2_O_3_ ETL. In this work, solvent-assisted crystallization of α-Fe_2_O_3_ ETLs was carried out to examine the impact of solvents on the optoelectronic properties of α-Fe_2_O_3_ thin films. Among the various solvents used in this study (deionized water, ethanol, iso-propanol, and iso-butanol), optimized ethanol-based α-Fe_2_O_3_ ETLs lead to champion device performance with a power conversion efficiency of 13% with a reduced hysteresis index of 0.04 in an n–i–p-configured PSC. The PSC also exhibited superior long-term inert and ambient stabilities compared to a reference device made using a SnO_2_ ETL. Through a series of experiments spanning structural, morphological, and optoelectronic properties of the various α-Fe_2_O_3_ thin films and their devices, we provide insights into the reasons for the improved photovoltaic performance. It is noted that the formation of a pinhole-free compact morphology of ETLs facilitates crack-free surface coverage of the perovskite film atop an α-Fe_2_O_3_ ETL, reduces interfacial recombination, and enhances charge transfer efficiency. This work opens up the route toward novel ETLs for the development of efficient and photo-stable PSCs.

## 1 Introduction

Hybrid inorganic–organic metal halide perovskite solar cells (PSCs) have emerged as a promising thin film photovoltaic (PV) technology due to their low cost of fabrication, high power conversion efficiencies (PCEs), and low-temperature processing.^1,2^ The unprecedented increase in device efficiencies from 3.8% in 2009 to a certified value of 25.7% in 2022 has instigated a revolution in PV technology.^3,4^ Such a high performance stems from the unique optoelectronic properties of perovskites such as a high absorption coefficient, long carrier lifetime, high mobility, and tunable band gap^5−7^ together with an extensive optimization of charge transport layers (CTLs), namely, the electron transport layer (ETL) and hole transport layer (HTL),^8,9^ and their interfaces with the perovskite layer. Controlling the properties of these CTLs is crucial as they control the physico-chemical properties of their interfaces with the perovskite and also control the extraction/transport of photogenerated charges to their respective electrodes.^10^ To achieve high device performance, the CTLs must exhibit low charge transfer resistance, minimal optical absorption, appropriate energy band level alignment with the absorber layer, and high mobility for efficient charge transport.^11−14^

In the state-of-the-art n–i–p PSCs, metal oxide (MO_X_) semiconductor TiO_2_-, ZnO-, SnO_2_-, Nb_2_O_5_-, CeO_*x*_*-*, Cr_2_O_3_-, Fe_2_O_3_-, and WO_3_-based ETLs remain a preferred choice.^15−22^ TiO_2_ and SnO_2_ are the most frequently applied ETLs, and solar cells based on these have demonstrated PCEs approaching 25%.^23−25^ The TiO_2_ ETL is known for its defect-rich surface and also leads to degradation in PSCs upon exposure to UV illumination hampering the device operational stability.^26,27^ The TiO_2_ ETL also requires a high annealing temperature exceeding 500 °C, which hinders its application in mass production. SnO_2_ ETLs can be processed at low temperatures and offer a high mobility, wide band gap, and superior stability than the TiO_2_ counterpart. Benefiting from these properties, SnO_2_ ETLs have also demonstrated PCE exceeding 25% performance in PSCs. Tin, however, suffers from limited supply issues which are reaching a critical level, and therefore, alternative non-toxic and abundant materials for ETLs should be explored.^28^ Likewise, ZnO is another n-type semiconductor metal oxide with high mobility, high transparency, and a wide band gap.^29^ Yet, the ZnO/perovskite interface induces chemical instability of the perovskite film owing to the presence of hydroxyl (−OH) groups.^30^

Recently, hematite (α-Fe_2_O_3_), a thermodynamically stable oxide of iron with n-type semiconducting properties, has attained global interest due to its low UV photocatalytic activity favorable in enhancing the UV stability of PSCs.^31^ α-Fe_2_O_3_ is a low-cost, abundant, and non-toxic material with an optical band gap of 2–2.3 eV.^32−34^ Due to its lower-lying conduction band minimum (CBM) than that of TiO_2_, α-Fe_2_O_3_ can expedite the electron extraction from the perovskite layer.^35^ However, a low mobility and poor conductivity of α-Fe_2_O_3_ in its pristine form limit its performance as the ETL in PSCs. Hu et al. employed α-Fe_2_O_3_ as the ETL in MAPbI_3_ PSCs and exhibited a PCE of 11% with improved stability as compared to a TiO_2_ ETL-based PSC. They reported a higher built-in potential across the perovskite layer in the α-Fe_2_O_3_ PSCs, which leads to enhanced charge extraction and lower charge accumulation at the interface.^36,37^ Similarly, Hou et al. employed a hematite–fullerene bilayer in a planar PSC and exhibited an enhanced PCE of 14%.^38^ Zhu et al. designed a non-equilibrium doping strategy to prepare Ti–Fe_2_O_3_ ETLs and exhibited a PCE of 17.8% with superior charge transport owing to high electron mobility and low trap density.^39^ Guo et al. applied interface engineering for bifunctional modification of the α-Fe_2_O_3_/perovskite interface by using PbI_2_ resulting in significant improvement in energy level alignment and suppressed *J*–*V* hysteresis.^20^ Yet, all these reports lack a detailed understanding of the morphology, crystallization, and optoelectronic properties of the pristine α-Fe_2_O_3_ ETL with their potential in PSCs and demand further investigation.

In this work, we report a systematic investigation of the solvent-assisted crystallization of hematite thin films using deionized water (DIW), ethanol, iso-propanol, and iso-butanol. The choice of the solvent used for crystallization impacts the film morphology, film thickness, and film quality.^40^ The triple-cation PSCs were fabricated in an n–i–p architecture to gain thorough insights into the impact of solvents on the PV performance of the α-Fe_2_O_3_ ETL-based devices. The PSCs with the optimized solvent and appropriate concentration demonstrated a PCE of 13.01%, which is till date the highest ever reported PCE obtained using a pristine α-Fe_2_O_3_ ETL. The optimal device also exhibited excellent long-term stability in the inert and ambient atmospheres as compared to a reference device based on a SnO_2_ ETL. The proposed strategy for optimizing the ETLs can be an important step forward for the other emerging ETLs for efficient and stable PSCs.

## 2 Experimental Section

### 2.1 Materials

Formamidinium iodide (FAI, 99.99%), methylammonium bromide (MABr, 99.99%) (Greatcell), lead(II)iodide (PbI_2_, 99.99%) (TCI), tin(IV)oxide (SnO_2_, 15% H_2_O colloidal dispersion) (Alfa Aesar), cesium iodide (CsI, 99.9%), bis(trifluoromethane sulfonyl)imide (Li-TFSI, 99.99%), iron(III)nitrate nonahydrate [Fe(NO_3_)_3_·9H_2_O, 99.9%], lead bromide (PbBr_2,_ 99.9%) (Sigma-Aldrich), indium tin oxide (ITO) substrates (ITO, 15 Ω/sq), and spiro-OMeTAD (>99%) (Lumtec) were used as procured. Dimethylformamide (DMF, 99.8%), 4-*tert*-butyl pyridine (tBP), chlorobenzene (CB, 99.8%), ethanol, isopropanol, dimethyl sulfoxide (DMSO, 99.9%), isobutanol, and acetonitrile (ACN, 99.9%) were purchased from Aldrich.

### 2.2 Preparation of ETL Dispersions

The ETL dispersions were prepared by dissolving Fe(NO_3_)_3_·9H_2_O in DIW, ethanol, isopropanol, and isobutanol in different mole ratios followed by stirring for 10 min as shown in Figure S1. The solutions were used to make ETLs under ambient conditions without filtering.

### 2.3 Preparation of Triple-Cation Perovskite and Spiro-OMeTAD Precursors

The triple-cation perovskite precursor was synthesized by dissolving 1.1 M PbI_2_ and 0.2 M PbBr_2_ in a mixed solvent of DMF/DMSO (4:1) followed by heating at 90 °C for 45 min. 1 M FAI and 0.2 M MABr were added to the above-mentioned solution. Finally, 53 μL of CsI (1.5 M CsI in DMSO) was mixed in the combined solution to attain the composition Cs_0.05_(FA_0.83_MA_0.17_)_0.95_Pb(I_0.83_Br_0.17_)_3_ of the triple-cation perovskite and/or for convenience as CsFAMA.^41^ To prepare spiro-OMeTAD (spiro) precursor solution, 73 mg of spiro was dissolved in CB (1 mL) followed by the addition of 17.5 μL of Li-TFSI (52 mg in 100 μL of ACN) and 28.8 μL of tBP as dopants to enhance conductivity.^42^

### 2.4 Device Fabrication

ITO-coated glass substrates were etched using zinc powder and HCl (2 M). The etched substrates were cleaned sequentially with soap, distilled water, acetone, and isopropyl alcohol for 20 min. The substrates were then dried using N_2_. The dried substrates were plasma-treated using O_2_ for 7 min followed by UV–ozone treatment for 20 min. The ETLs were prepared by spin-coating the Fe(NO_3_)_3_·9H_2_O dispersions (75 μL) in different solvents (DIW, ethanol, iso-propanol, and iso-butanol) and with molar concentrations (0.1, 0.2, 0.5, and 1 M) at 4000 rpm (1000 rpm s^–1^) for 45 s followed by thermal annealing at 300 °C for 60 min with a ramp rate of 10°/min. The ETL-deposited substrates were transferred to a N_2_-filled glovebox to deposit the perovskite and HTL. The perovskite precursor (45 μL) was spin-coated on the ITO/α-Fe_2_O_3_ substrate at 1000 rpm (1000 rpm s^–1^) for 10 s and then 6000 rpm (4000 rpm s^–1^) for 25 s followed by antisolvent CB (250 μL) dripping for 10 s before the end of the second step, followed by annealing at 120 °C for 10 min. The HTLs were prepared by spin-coating 30 μL of the spiro precursor onto the perovskite layer at 4000 rpm (1000 rpm s^–1^) for 40 s. The HTL-deposited films were stored in a desiccator for 12 h before electrode evaporation. Finally, WO_3_ (3 nm) and Ag (100 nm) were thermally evaporated under a vacuum of 8 × 10^–6^ mbar. The active area of the PSCs was defined by a shadow mask to be 0.133 cm^2^.

### 2.5 Characterization

X-ray diffraction (XRD) patterns were recorded to examine the crystalline structure of prepared samples using an X-ray diffractometer (Bruker D8 ADVANCE). The morphology of the thin films was investigated by field-emission scanning electron microscopy (FE-SEM, Zeiss Gemini). The topography of the perovskite thin films was further analyzed by atomic force microscopy (Park NX 10). The optical characteristics were investigated by measuring transmission and absorption spectra via a UV–vis–NIR spectrophotometer (Cary 5000). The steady-state photoluminescence (PL) and time-resolved PL (TRPL) measurements were performed to study the charge transport mechanism using a fluorescence spectrometer (PicoQuant FluoTime 300) equipped with a 404 nm laser source. The PSCs were measured inside the glovebox for photocurrent–voltage (*J*–*V*) measurements using a Keithley 2410 source meter equipped with a solar simulator under a simulated AM 1.5 G spectrum (100 mW cm^–2^). The external quantum efficiency measurements (EQE) were performed by using a xenon light source equipped with a grating monochromator (LOT-Oriel Omni 300). The carrier mobility and trap densities were measured using space charge limited current (SCLC) measurements.

## 3 Results and Discussion

Formation of a high-quality ETL strongly depends on the solvent used for the hydrolysis and the annealing process, among other important parameters.^40^ For our work, we have selected four types of solvents, i.e., DIW (evaporation point 100 °C), ethanol (78 °C), iso-propanol (82 °C), and iso-butanol (108 °C), for the crystallization of α-Fe_2_O_3_ ETLs. These various α-Fe_2_O_3_ ETLs are labeled as W-α-Fe_2_O_3_, E-α-Fe_2_O_3_, iP-α-Fe_2_O_3_, and iB-α-Fe_2_O_3_, respectively. The α-Fe_2_O_3_ ETLs were prepared by spin-coating an iron nitrate nonahydrate [Fe(NO_3_)_3_·9H_2_O] precursor dispersed in different solvents (Figure S1) at 4000 rpm for 45 s followed by thermal annealing at 300 °C for 60 min in air.

**Figure 1:**
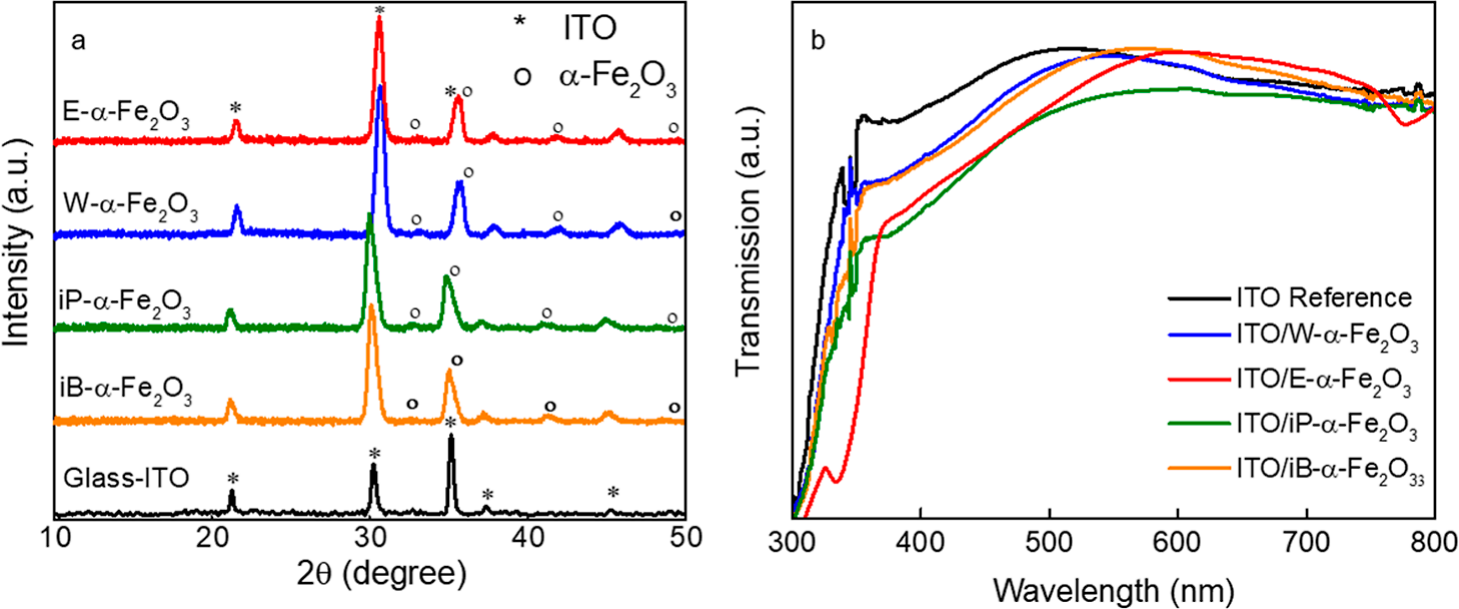
(a) XRD spectra of α-Fe_2_O_3_ thin films with different solvents used for crystallization and (b) transmission spectra of the same.

Figure 1a shows the XRD patterns of the different α-Fe_2_O_3_ ETLs deposited on ITO. The diffraction peaks at 33.1, 35.8, 41, and 49.1° can be assigned to (104), (110), (113), and (024) crystallographic planes of rhombohedral hematite, respectively (JCPDS: 01-1053).^43^ The XRD diffractograms suggest identical crystallinities for the ETLs prepared from different solvents. Transmittance spectra of the various α-Fe_2_O_3_ ETLs (Figure 1b. See absorbance data in Figure S2) deposited over the ITO surface show variation in the transmittance for different solvents used, which is the result of different film morphologies and different thicknesses. The lowest transmittance for the iP-α-Fe_2_O_3_ ETL in the entire wavelength range is due to a higher film thickness of the ETL, which can have detrimental impact on the light management in the final device stack.^44^ The onset of the transmittance, suggest that the different solvents used for crystallization can also lead to different stoichiometry or defect density leading to a different optical band gap of the α-Fe_2_O_3_ films.

**Figure 2:**
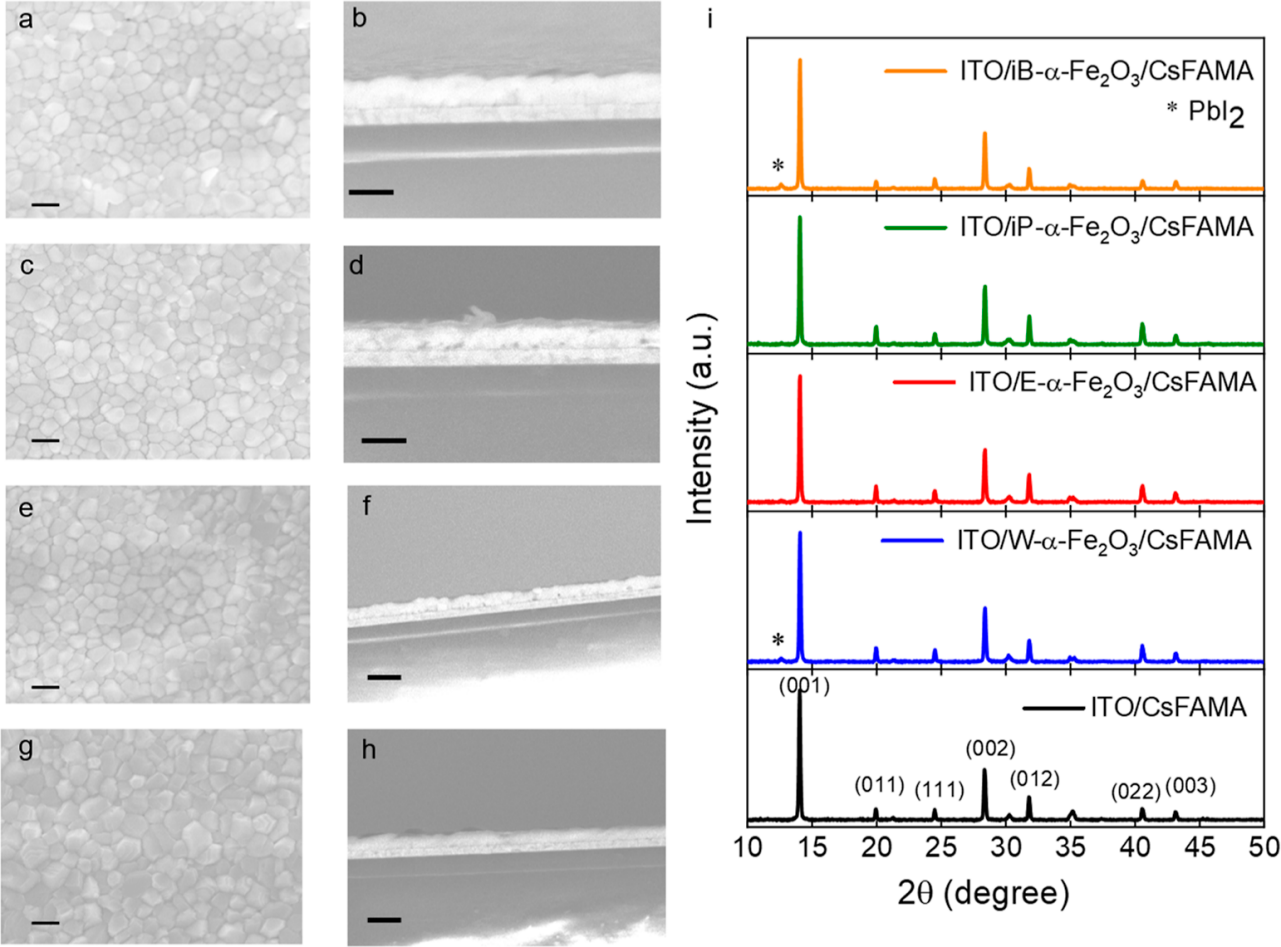
Top and cross-sectional views of CsFAMA perovskite thin films deposited atop ITO/α-Fe_2_O_3_ ETLs: (a,b) W-α-Fe_2_O_3_, (c,d) E-α-Fe_2_O_3_, (e,f) iP-α-Fe_2_O_3_, and (g,h) iB-α-Fe_2_O_3_. (i) XRD spectrum of CsFAMA perovskite thin films deposited on top of the different α-Fe_2_O_3_ ETLs.

The surface morphology of different α-Fe_2_O_3_ ETLs deposited on glass/ITO substrates was analyzed by SEM and was compared with the morphology of a bare ITO (Figure S3). The various α-Fe_2_O_3_ ETLs adapt the ITO surface morphology owing to the formation of an ultra-thin layer. Clearly, the choice of the solvent plays an important role in determining the morphology of the ETL. While all the ETLs show a good coverage over the ITO substrate, the E-α-Fe_2_O_3_ and iP-α-Fe_2_O_3_ ETLs show the least ITO patches. This difference in the morphology can originate from different film thicknesses due to the different solvents used. A higher film thickness for E-α-Fe_2_O_3_ and iP-α-Fe_2_O_3_ ETLs is evident from the transmission spectra, which also improves the surface coverage of these ETLs. Figure 2 exhibits the top and cross-sectional views of the triple-cation perovskite (CsFAMA) deposited on the various α-Fe_2_O_3_ ETLs. All ETLs demonstrate a crack-free, uniform, and densely packed morphology. The cross-sectional SEM images of perovskite films suggest fused perovskite grain boundaries, which are favorable for efficient charge transport and low non-radiative recombination.^45^ The crystallinity of the CsFAMA perovskite deposited onto the W-α-Fe_2_O_3_, E-α-Fe_2_O_3_, iP--α-Fe_2_O_3_, and iB-α-Fe_2_O_3_ ETLs and bare ITO is depicted in Figure 2i. All perovskite films show preferential crystal orientation for the (001) and (002) planes. No peak associated with the hexagonal δ-phase was observed for the perovskite films onto α-Fe_2_O_3_ ETLs confirming the formation of a desirable tetragonal photoactive α-phase (black).^46^ Notably, a diffraction peak at 2θ position of 12.6° was observed for W-α-Fe_2_O_3_ and iB-α-Fe_2_O_3_ ETLs, which suggests a PbI_2_ residue probably due to incomplete conversion of the perovskite phase or due to degradation of the perovskite film over these ETLs.

**Figure 3:**
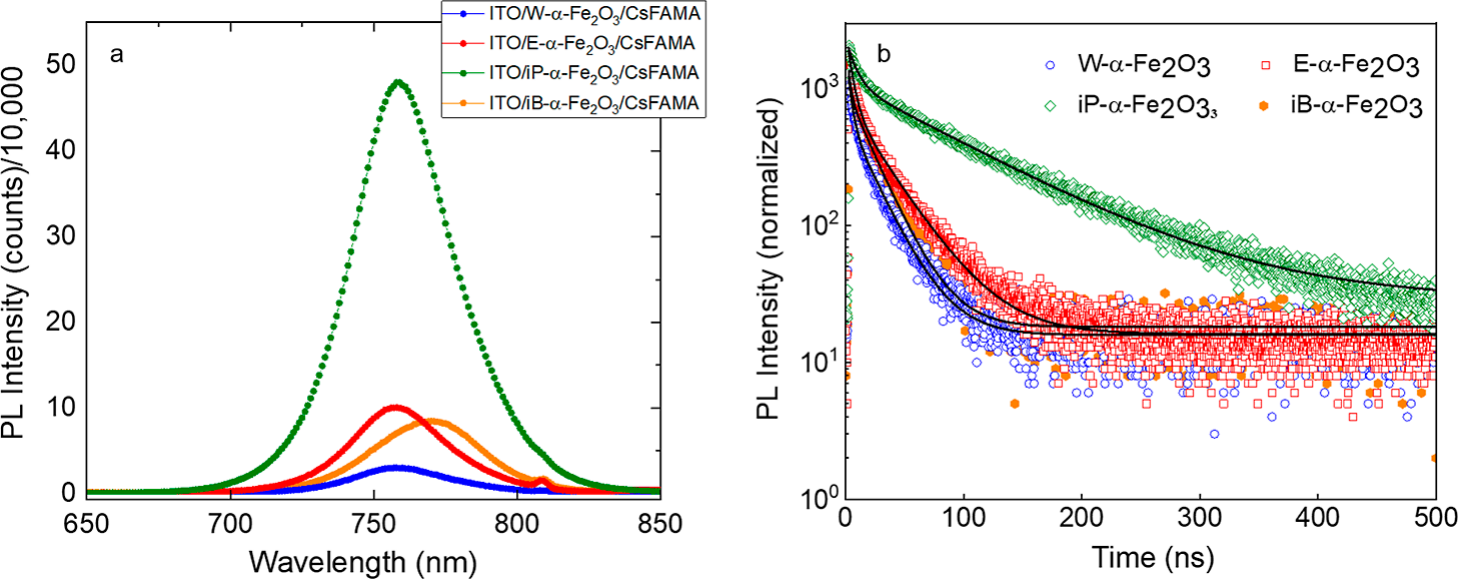
(a) Steady-state PL spectra of CsFAMA films on the ITO/α-Fe_2_O_3_ ETL with different solvents and (b) TRPL transients of the same. The PL is recorded using a pulsed laser with an excitation wavelength of 404 nm and a repetition rate of 2 MHz.

In order to probe the charge extraction rates from the perovskite layer to the various ETLs, we recorded steady-state PL and TRPL spectra of CsFAMA perovskite layers deposited atop W-α-Fe_2_O_3_, E-α-Fe_2_O_3_, iP-α-Fe_2_O_3_, and iB-α-Fe_2_O_3_ ETLs (Figure 3). Initially, the CsFAMA deposited on the bare ITO glass substrate exhibited an emission peak centered at 761 nm with a τ_ave_ of 238 ns (Figure S4 and Table S1). The CsFAMA deposited on the W-α-Fe_2_O_3_, E-α-Fe_2_O_3_, and iP-α-Fe_2_O_3_ ETLs shows suppressed PL emission intensity, which is attributed to quenching of charge carriers. A comparison of the PL spectra suggests nearly 5 times higher PL intensity for the iP-α-Fe_2_O_3_ ETL than that for other ETLs, which is indicative of inefficient charge transfer. The quenched PL intensity for W-α-Fe_2_O_3_, E-α-Fe_2_O_3_, and iB-α-Fe_2_O_3_ ETL samples indicates a more efficient charge carrier extraction and transport from the perovskite layer to the aforementioned ETLs. One should, however, note that the PL spectra contain information about two processes, i.e., charge extraction and interfacial recombination, and lower (or higher) PL spectra can have a contribution from both factors. From a PL spectrum on a quenching surface alone, such as the one used in this study, a distinction between the two processes cannot be made. Comparing the PL spectra with TRPL transients, to some extent, provides a more accurate overview of the interfacial processes.

To further examine the charge transport kinetics, the TRPL decay transients were fitted using a bi-exponential function consisting of τ_1_ and τ_2_ (related to fast and slow decay components, respectively) and A (decay amplitude)
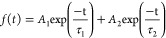
1and average lifetime was calculated according to eq 2 and results are summarized in Table S2.
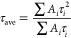
2

The fast decay component is attributed to charge carrier extraction by the ETL and non-radiative recombination (within the bulk or at the perovskite surface/interface), while the slow decay component is related to radiative recombination of the charge carriers in the bulk of the perovskite films.^41^ Fitting the TRPL transients yielded a τ_1_ of 3.40, 4.82, 7.58, and 2.22 ns for W-α-Fe_2_O_3_, E-α-Fe_2_O_3_, iP-α-Fe_2_O_3_, and iB-α-Fe_2_O_3_ ETLs with an amplitude of 69.2, 70.7, 52.9, and 62.72%, respectively. The higher amplitudes and slow τ_1_ for DIW and ethanol solvent-based ETLs suggest faster electron extraction, which may be due to a lower trap density in the perovskite films deposited atop these ETLs. Notably, the iB-α-Fe_2_O_3_ ETL sample showed the smallest τ_1_, suggesting the least efficient charge extraction from the perovskite among all the four ETLs. This is affirmed from the red-shifted PL emission peak that also suggests a higher defect density in the iB-α-Fe_2_O_3_/perovskite and also the lowest PCE in devices, as will be discussed later on. It is important to note that a reduced lifetime on a quenching film could originate from either a better charge transfer efficiency or a higher defect density at the interface. TRPL transients alone cannot decouple the two possible mechanisms, and one has to also compare the device performances to suggest a more plausible scenario. The average lifetime for the W-α-Fe_2_O_3_, E-α-Fe_2_O_3_, and iB-α-Fe_2_O_3_ ETLs is lower than that for the iP-α-Fe_2_O_3_ ETL. A comparison of the PL lifetimes of the various ETLs with their PL intensities suggests that the iP-α-Fe_2_O_3_ ETL leads to the least efficient charge extraction than the other three ETLs.

**Figure 4:**
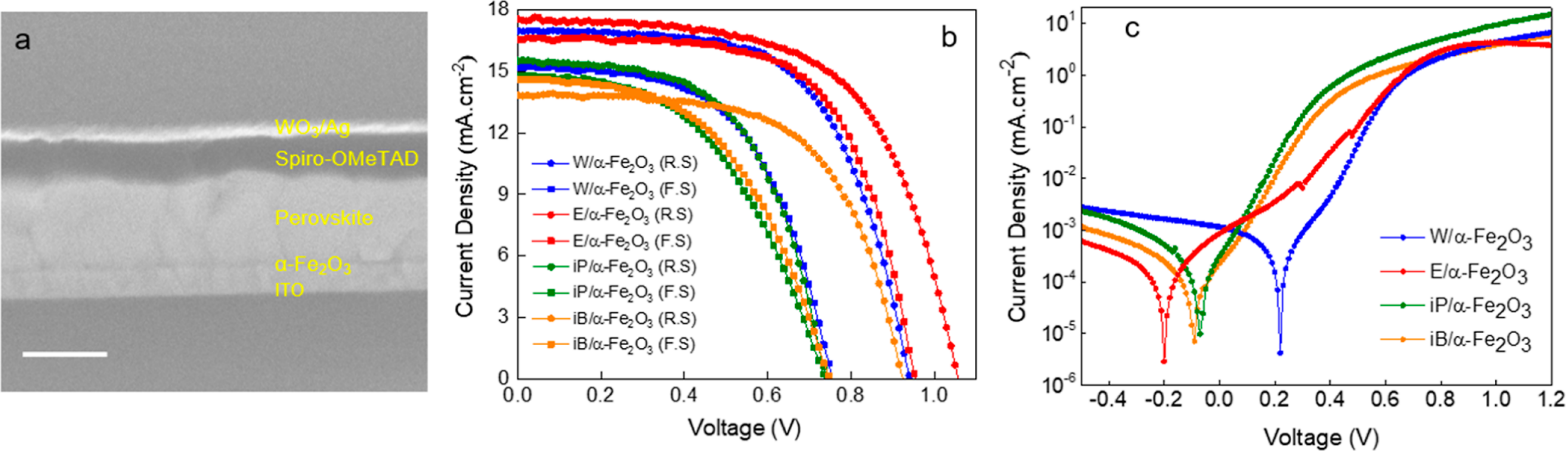
(a) Cross-sectional view of a complete PSC. Scale bar 400 nm. (b) *J*–*V* curves of the PSCs using four different α-Fe_2_O_3_ ETLs in forward and reverse scan directions. (c) Dark *J*–*V* curves of the same.

In order to validate the charge transfer behavior of these various ETLs, we fabricated PSCs in regular planar (n–i–p) configuration with the structure (ITO/α-Fe_2_O_3_/CsFAMA/spiro-OMeTAD/WO_3_/Ag) as shown in Figure 4a. The cross-sectional view shows a crack-free dense perovskite film of thickness around 400 nm deposited atop the α-Fe_2_O_3_ ETL.

The PV performance of PSCs was measured under 1 sun illumination AM 1.5G irradiation at a scan rate of 0.01 V s^–1^. The corresponding PV parameters, i.e., the open-circuit voltage (*V*_OC_), short current density (*J*_SC_), and fill factor (FF), together with the PCE in the forward scan (*J*_SC_ to *V*_OC_) and reverse scan (*V*_OC_ to *J*_SC_) are summarized in Figure 4b and Table 1. The control device was fabricated using the SnO_2_-based ETL (Figure S5). We also calculated the hysteresis index (HI) using the relation HI = PCE_R_ – PCE_F_/PCE_R_^47^ to compare the hysteresis in various devices. The W-α-Fe_2_O_3_, iP-α-Fe_2_O_3_, and iB-α-Fe_2_O_3_ ETL-based PSCs exhibited a PCE of 8.62%, 5.68, and 7.02%, respectively. The E-α-Fe_2_O_3_ ETL-based device exhibited the highest PCE of 10.07% (*J*_SC_ of 15.67 mA cm^–2^, *V*_OC_ of 1.06 V, and FF of 60.8%) and also showed the lowest HI of 0.10 (Table 1). The superior performance in the E-α-Fe_2_O_3_-based PSC stems mostly from an improved *V*_OC_ (130 mV higher than that of the W-α-Fe_2_O_3_, which showed the second highest PCE) and FF, both suggesting reduced interfacial recombination for this type of ETL. This is also confirmed from the reduced hysteresis—a phenomenon that strongly depends on the recombination and interfacial charge accumulation of the E-α-Fe_2_O_3_-based PSCs (HI of 0.1) than all other ETLs.^44,48^

**Table 1:** Photovoltaic Parameters of PSCs Using α-Fe_2_O_3_ Made from Different Solvents

ETL	scan direction	*J*_SC_ (mA cm^–2^)	*V*_OC_ (V)	FF (%)	PCE (%)	H.I.
W-α-Fe_2_O_3_	reverse	16.99	0.93	61.48	9.71	0.35
	forward	15.17	0.74	56.54	6.35	
E-α-Fe_2_O_3_	reverse	17.55	1.06	60.88	11.33	0.11
	forward	16.56	0.95	64.16	10.09	
iP-α-Fe_2_O_3_	reverse	15.51	0.73	56.40	6.39	0.19
	forward	14.80	0.72	48.57	5.18	
iB-α-Fe_2_O_3_	reverse	13.84	0.92	62.08	7.90	0.32
	forward	14.62	0.73	50.64	5.40	
SnO_2_	reverse	19.03	1.14	76.22	16.60	0.10
	forward	17.99	1.12	73.03	14.79	

All these observations suggest that the E-α-Fe_2_O_3_ ETL forms a favorable interface with the perovskite layer. The results are also consistent with PL and TRPL measurements that show a superior charge extraction using the E-α-Fe_2_O_3_ ETL. A higher HI in the other ETLs is indicative of higher interfacial recombination, thus rendering them not favorable for high-efficiency PSCs. The control device with the SnO_2_ ETL exhibited a PCE of 16.6%. Although the PCE of the α-Fe_2_O_3_ ETL is lower than that of the SnO_2_ counterpart, the fact that the low-cost hematite shows a comparable performance in PSC shows potential as alternative ETL. We also investigated the PV performance of the PSC based on α-Fe_2_O_3_ ETLs using different solvents (DIW, iso-propanol, and iso-butanol) in varying concentrations. The corresponding *J*–*V* curves are shown in Figure S6, and the parameters are listed in Table S3. A comparison of this data together with Tables 1 and 2 suggests ethanol to be a preferred solvent for the crystallization of hematite thin films, which results in the highest PV performance parameters. The E-α-Fe_2_O_3_ ETL-based films manifested a high-quality and pin-hole-free compact ETL. This is probably due to the better solubility of the α-Fe_2_O_3_ precursor in ethanol, which originates from the presence of the hydroxyl group and a polar C–O bond making its interaction with other materials easier.

**Table 2:** Photovoltaic Parameters of the PSCs Made Using E-α-Fe_2_O_3_ ETLs with Different Molar Concentrations

conc. (M)	scan direction	*J*_SC_ (mA cm^–2^)	*V*_OC_ (V)	FF (%)	PCE (%)	H.I.
0.1	reverse	17.56	1.06	60.88	11.33	0.11
	forward	16.56	0.95	64.16	10.09	
0.2	reverse	19.21	1.05	64.48	13.01	0.04
	forward	18.37	1.05	64.99	12.54	
0.5	reverse	13.91	0.8	67.05	7.46	0.17
	forward	11.83	0.78	66.71	6.16	
1	reverse	11.78	0.72	58.81	4.99	0.19
	forward	10.61	0.64	59.42	4.03	

The dark *JV* curves of these devices provide further insights into the origin of varying PV performance due to the different ETLs (Figure 4c). The E-α-Fe_2_O_3_ ETL shows the lowest reverse current among all the ETLs which indicates its efficient hole blocking behavior. This possibly originates from the improved morphology of the E-α-Fe_2_O_3_ ETL. The iP-α-Fe_2_O_3_ and iB-α-Fe_2_O_3_ ETL-based devices demonstrated a higher reverse current density suggesting that higher leakage currents in these devices limit the PV performance.

We chose the E-α-Fe_2_O_3_ ETL for further investigation and optimization. We first optimized the molar concentrations of E-α-Fe_2_O_3_ in a wide range of 0.1, 0.2, 0.5, and 1 M to vary the film thickness. The transmission spectra for the various E-α-Fe_2_O_3_ ETLs deposited on ITO show a decrease in the transmittance with increasing molar concentrations suggesting the formation of a thicker ETL (Figure S7). The absorption spectra exhibited identical absorption in wavelengths ranging from 550 to 800 nm (Figure S8). The top and cross-sectional SEM images of ITO/E-α-Fe_2_O_3_ ETLs with concentrations (of 0.2, 0.5, and 1 M) confirm a uniform and continuous surface coverage with no visible pinholes (Figure S9). The thickness of E-α-Fe_2_O_3_ ETLs with concentrations of 0.2, 0.5, and 1 M is found to be 42, 68, and 127 nm, respectively. One should note the trade-off between the film morphology and transmittance as the film thickness varies. While a thinner layer improves the transmittance of the ultrathin ETLs, it often yields films with pinholes due to incomplete surface coverage. A thick ETL although facilitates the formation of a pinhole-free film but at the cost of reduced transmittance, which limits the light absorption efficiency of the perovskite layer and the attainable *J*_SC_ thereby. Ideally, a thin compact ETL with negligible transmittance is preferred.

**Figure 5:**
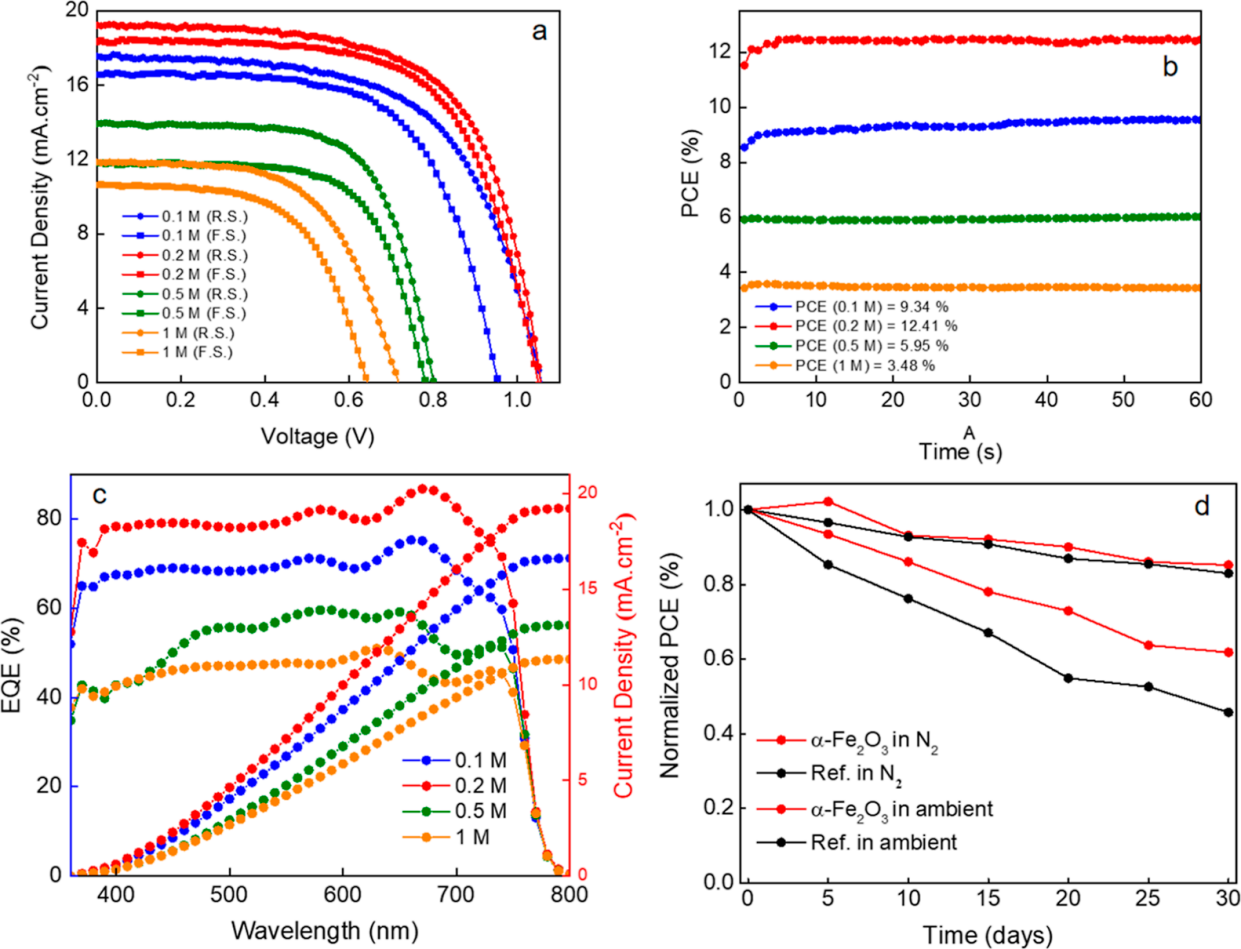
(a) *J*–*V* curves of the PSCs using E-α-Fe_2_O_3_ ETLs of different molar concentrations, (b) maximum power point tracking and (c) EQE spectra of the same, and (d) shelf-life measurements of the champion and control devices measured under the same experimental conditions.

We investigated the PV performance of these various ETLs in PSCs (Figure 5a), and the corresponding PV parameters are summarized in Table 2. The 0.2 M E-α-Fe_2_O_3_ ETL-based device exhibited the highest PCE of 13% (*J*_SC_ of 19.2 mA cm^–2^, *V*_OC_ of 1.05 V, and FF of 64.48%) with an HI of 0.04. An optimized ETL thickness is necessary. While thinner ETLs increasing charge collection also lead to higher recombination, we observe a reduced charge collection for thick films, again leading to higher recombination, which impacts the *J*_SC_ and FF. As can be seen in Table 2, in the case of a thinner ETL (0.1 M concentration), we observed an HI of 0.1 and a lower *J*_SC_ than that of the 0.2 M counterpart, which is indicative of higher recombination as the ETL thickness decreased. The superior performance of the 0.2 M concentration was reproducible over several devices fabricated in different batches, as shown in Figure S10.

We further recorded dark *JV* measurements of the PSCs (Figure S11), which also exhibited suppressed charge recombination for the optimized 0.2 M E-α-Fe_2_O_3_ ETL-based PSC and the highest injection current. Notably, the optimized 0.2 M E-α-Fe_2_O_3_ ETL-based PSCs showed slightly lower performance than a control device made using the SnO_2_ ETL (PCE of 16.60%) suggesting room for further improvement. Nevertheless, these results show that with an optimized morphology and thickness, low-cost hematite has high potential as a future electron transport material.

For a reliable reporting of PCE, we measured stabilized PCE using maximum power point tracking (Figure 5b). The stabilized PCEs are 9.34, 12.4, 5.9, and 3.5% for 0.1, 0.2, 0.5, and 1 M E-α-Fe_2_O_3_ ETLs, respectively. To validate the *J*_SC_ in our PSCs, we measured EQE. Figure 5c demonstrates the spectra for the perovskite devices with E-α-Fe_2_O_3_ ETLs. The highest EQE for the 0.2 M concentration affirms optimized charge collection using this ETL. The integrated *J*_SC_ calculated from the EQE spectra matches well with that obtained from *J*–*V* measurements confirming the reliability of the measurements performed. In order to investigate the stability of the α-Fe_2_O_3_ ETL-based PSCs, we measured device stability of the champion and control devices in an inert (N_2_) and in an ambient atmosphere for 30 days (Figure 5d). The 0.2 M E-α-Fe_2_O_3_ ETL-based device demonstrated comparable stability to the SnO_2_ reference counterpart in an inert atmosphere and retained 86% of its initial PCE after 30 days of storage. For the devices stored under ambient conditions, superior stability is noted for the 0.2 M E-α-Fe_2_O_3_ ETL-based PSC, which retained 62% of the initial PCE (higher than the reference PSC showing only 45% of the initial PCE).

**Figure 6:**
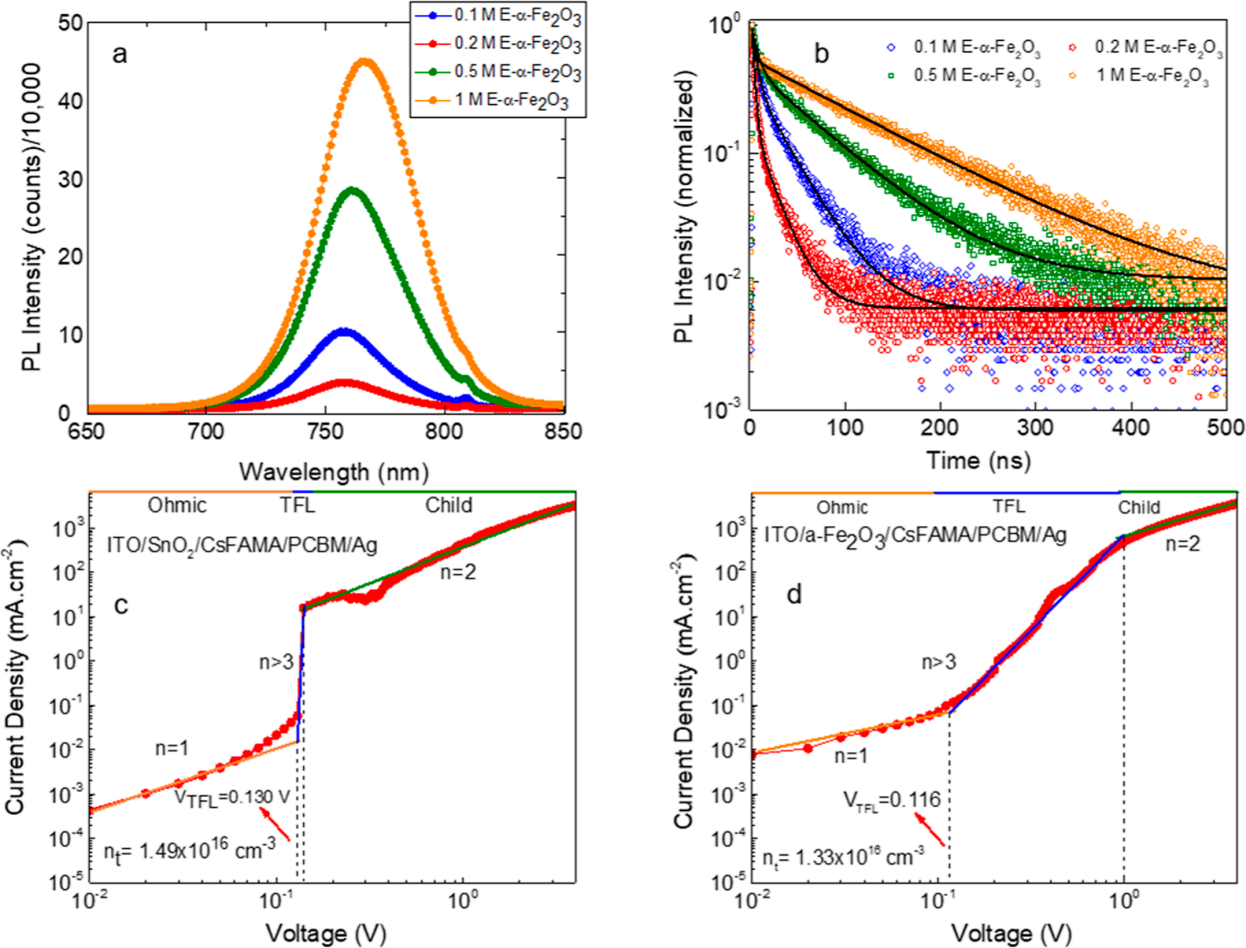
(a) Steady-state PL spectra and (b) time-resolved PL transients of CsFAMA films deposited on ITO/E-α-Fe_2_O_3_ ETLs with different molar concentrations in ethanol. (c) SCLC curve of a reference ETL made using SnO_2_ vs (d) SCLC curve of the champion ETL.

In order to investigate the mechanism behind the improved PV performance of the 0.2 M E-α-Fe_2_O_3_ ETL-based PSCs, we performed PL and TRPL measurements. Figure 6a shows the PL spectra for the CsFAMA perovskite deposited on the various E-α-Fe_2_O_3_ ETLs with different molar concentrations. The films were deposited on ITO glass and excited from the glass side (ETL/perovskite interface) to better compare the properties at this interface. The 0.2 M E-α-Fe_2_O_3_ ETL shows around 3, 8, and 12 times quenched PL intensity than 0.1, 0.5, and 1 M counterparts, respectively, and also a blue-shifted PL emission peak. The emission peaks for the perovskite films deposited atop 0.1, 0.2, 0.5, and 1 M 0.2 M E-α-Fe_2_O_3_ ETLs are centered at 756, 757, 760, and 766 nm, respectively, suggesting a lower defect density in the perovskite films on 0.1 and 0.2 M ETLs. The TRPL measurements also affirm a faster charge extraction in the 0.2 M sample. In order to extract the carrier extraction time, the TRPL transients were fitted using a biexponential function (Figure 6b), and the corresponding parameters are listed in Table S4. The 0.2 M E-α-Fe_2_O_3_ ETL showed the fastest τ_1_ of 2.55 s with an amplitude of 90.87%, which together with the *JV* data shown in Figure 5 and Table 2 (the highest *J*_SC_ and EQE for 0.2 M films) suggests an enhanced charge transfer efficiency at the ETL/perovskite interface using this ETL. The average lifetime also showed a strong dependence on the ETL concentration, and the shortest τ_average_ of 10 ns was noted for the optimal 0.2 M ETL, which is manifolds faster than that of its counterparts.

To confirm the reduced defect density (*n*_*t*_) in the optimal ETL, we performed the SCLC measurements in reference (ITO/SnO_2_/CsFAMA/PCBM/Ag) and target ETL-based devices [ITO/E-α-Fe_2_O_3_ (0.2 M)/CsFAMA/PCBM/Ag], Figure 6c,d. The SCLC curve consists of three distinct regions (Ohmic, trap-filled, and trap-free) based on the value of the exponential (*n*).^49,50^ The conductivities (σ) of crystals can be assessed in the Ohmic region at a low bias (*I* ∝ *V*). The charge carrier mobilities can be evaluated in the trap-free region at a high bias (*n* = 2, cyan line) by Mott–Gurney’s law ().^51^ The third region where the current rapidly increases is called the trap-filling region (*n* > 3, pink line), which is used to measure the trap densities as  where *V*_TFL_ is trap-filled limit voltage, *L* is the thickness of the absorber layer, ε is the dielectric constant (ε = 65 for the perovskite), ε_0_ is the vacuum permittivity (8.8542 × 10^–14^ F/cm), and *e* is the charge (1.602 × 10^–19^ C).^17^ The trap densities were calculated to be 1.33 × 10^16^ cm^–3^ and 1.49 × 10^16^ cm^–3^ for the target and reference devices, respectively. A lesser trap density for the target device suggests fewer defect states as compared to the reference device. Similarly, the electron mobility was measured by fabricating the electron-only devices with configuration (ITO/E-α-Fe_2_O_3_/WO_3_/Ag) using the concentrations 0.1 and 0.2 M as shown in Figure S15. The values for mobility are found to be 3.7 × 10^–4^ cm^2^ V s^–1^ and 5.2 × 10^–4^ cm^2^ V s^–1^ for 0.1 and 0.2 M E-α-Fe_2_O_3_ ETLs, respectively. The mobility values are consistent with the previous literature^52^ suggesting that the films made in this work are of high quality.

## 4 Conclusions

In summary, the solvent-assisted crystallization of α-Fe_2_O_3_ thin films with various solvents (DIW, ethanol, iso-propanol, and iso-butanol) has been investigated thoroughly. Our experiments revealed that the choice of the solvent significantly impacts the morphology and defect density of the α-Fe_2_O_3_ ETLs. The ethanol-based ETLs show a pinhole-free compact film formation, which results in improved charge extraction and superior PV performance than those of the ETL prepared using other solvents. The improved performance also stems from a superior hole-blocking capability of the E-α-Fe_2_O_3_ ETL, as evident from the dark current–voltage curve comparison. The optimized E-α-Fe_2_O_3_ ETL demonstrated a pinhole-free compact morphology facilitating the formation of a high-quality crack-free perovskite film atop. When compared to a SnO_2_ reference, the E-α-Fe_2_O_3_ ETL also showed a lower trap density, high electron transfer efficiency, and electron mobility—all these contributing to a PCE of 13% with a low HI of 0.04. The target device also exhibited long-term stability in an inert and ambient environment as well, thus showing the potential of α-Fe_2_O_3_ ETLs for the development of efficient and stable PSCs.

## Supporting Information Available

Characterization data, device parameters of reference and Fe_2_O_3_-containing devices, statistical analysis of device performance parameters, atomic force microscopy and SEM images of perovskite films deposited over ETLs, dark *JV* curves, and SCLC curves (PDF)
