# Supplementary material for: Solvent-Assisted Crystallization of an α-Fe2O3 Electron Transport Layer for Efficient and Stable Perovskite Solar Cells Featuring Negligible Hysteresis
Source: ACS Omega. 2023 May 9;8(20):18106–15. doi: 10.1021/acsomega.3c01405 (PMC10210035; doi:10.1021/acsomega.3c01405)
Supplement: Supplementary file 1 — ao3c01405_si_001.pdf [file ao3c01405_si_001.pdf]

Solvent-Assisted Crystallization of α-Fe_2O_3 Electron Transport Layer for Efficient and Stable Perovskite Solar Cells Featuring Negligible Hysteresis

Akbar Ali Qureshi [a, b, c], Sofia Javed* [a,] Muhammad Aftab Akram [d], Lukas Schmidt-Mende* [c], Azhar Fakharuddin* [c]

[a] School of Chemical & Materials Engineering, National University of Sciences & Technology, 44000, Islamabad, Pakistan

[b] Department of Mechanical Engineering, Bahauddin Zakariya University, 60000, Multan, Pakistan

[c] Department of Physics, University of Konstanz, 78464, Konstanz, Germany

[d] Department of Materials Science & Engineering, Pak-Austria Fachhochschule, Institute of Applied Sciences & Technology, 22650, Haripur, Pakistan

Correspondce: SJ (sofia.javed@scme.nust.edu.pk), LSM (Lukas.Schmidt-Mende@uni-konstanz.de), AF (Lukas.Schmidt-Mende@uni-konstanz.de).

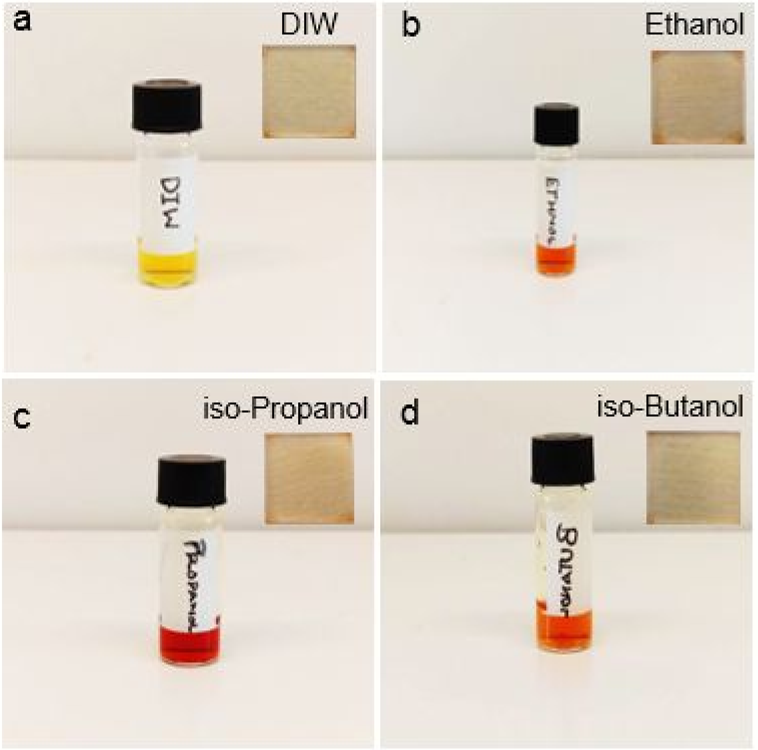

Figure S1. α-Fe_2O_3 dispersion in different solvents: (a) deionized water, (b) ethanol, (c) iso-propanol, and (d) iso-butanol. (Inset shows thin films annealed @ 300 °C for each solvent)

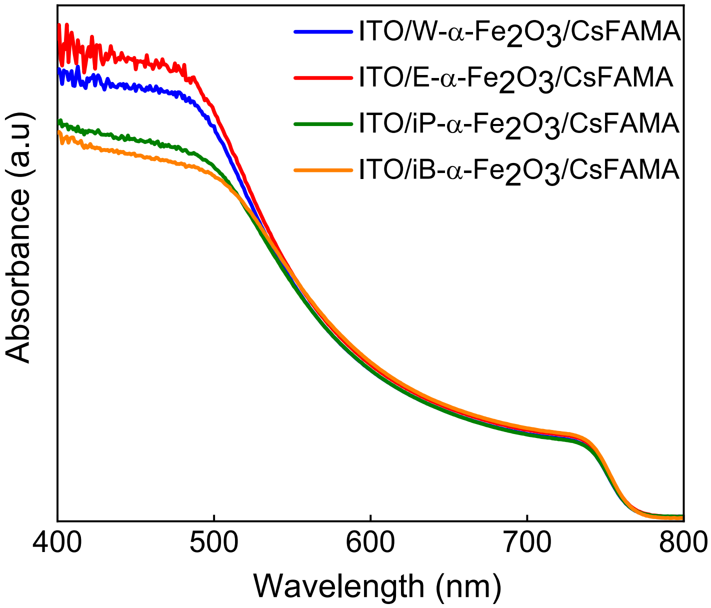

Figure S2. Absorption spectra of CsFAMA films on ITO/α-Fe_2O_3 ETL with different solvents,

Figure S4 exhibits the absorption spectra of CsFAMA onto W-α-Fe_2O_3, E-α-Fe_2O_3, iP-α-Fe_2O_3, and iB-α-Fe_2O_3, ETLs. The spectra exhibited almost identical absorption over the spectral range of 500 to 800 nm for different α-Fe_2O_3 ETLs. The E-α-Fe_2O_3 and W-α-Fe_2O_3 ETLs manifested significantly enhanced absorption in the spectral range of 400 to 500 nm as compared to other ETLs. The slight difference in shorter wavelengths can be due to parasitical absorption or dense surface coverage.

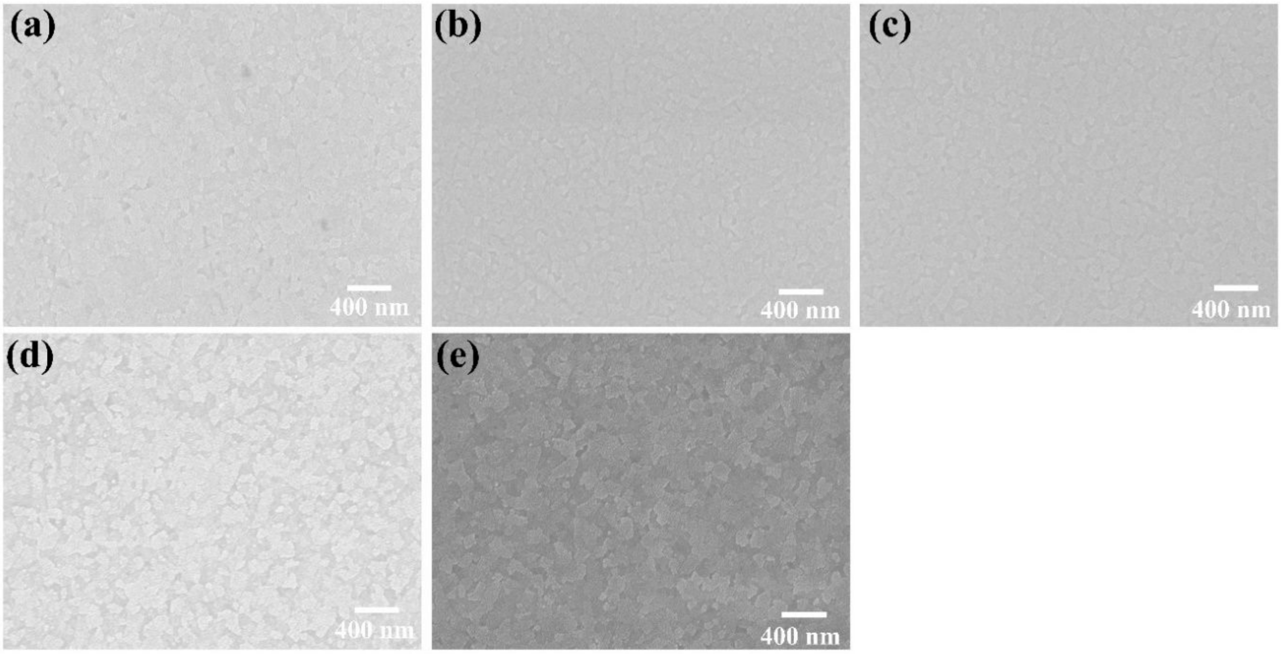

Figure S3. Top view SEM image of ITO/α-Fe_2O_3 thin films in different solvents: (a) W-α-Fe_2O_3, (b) E-α-Fe_2O_3, (c) iP-α-Fe_2O_3, (d) iB-α-Fe_2O_3, and (e) pristine ITO.

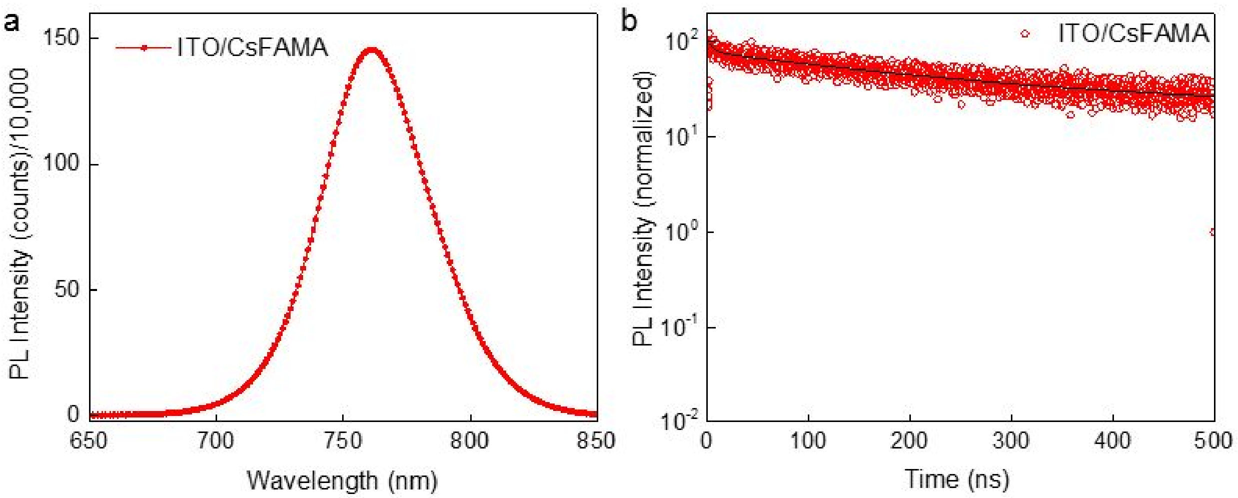

Figure S4. (a) Steady-state PL spectra of CsFAMA film on ITO substrate, and (b) time-resolved PL transients of the same.

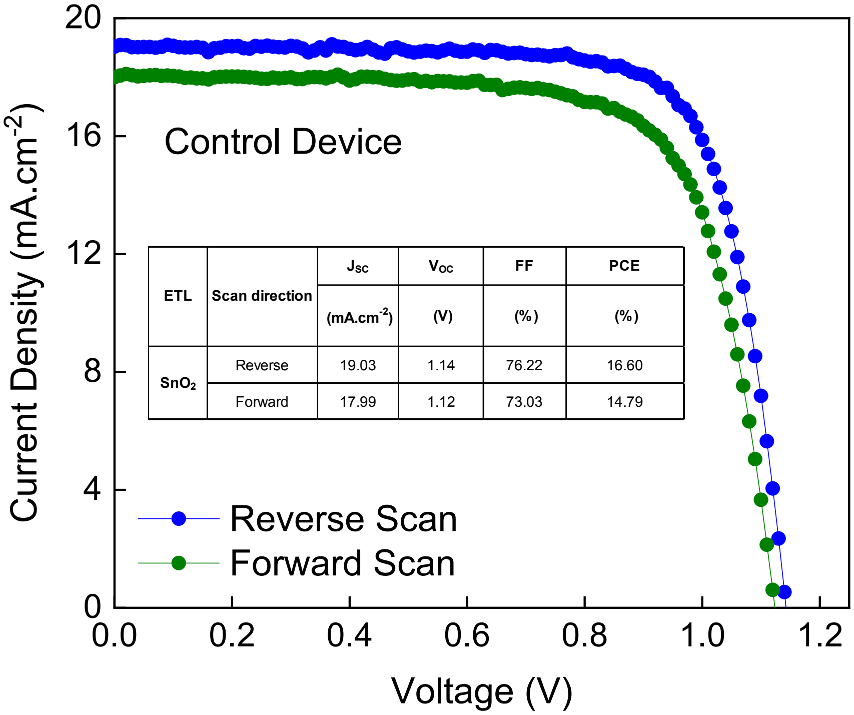

ETL	Scan direction	J_{SC} ($mA.cm^{-2}$)	V_{OC} (V)	FF (%)	PCE (%)
SnO_2	Reverse	19.03	1.14	76.22	16.60
	Forward	17.99	1.12	73.03	14.79

Figure S2. Light J-V curves of a control device with SnO_2 ETL.

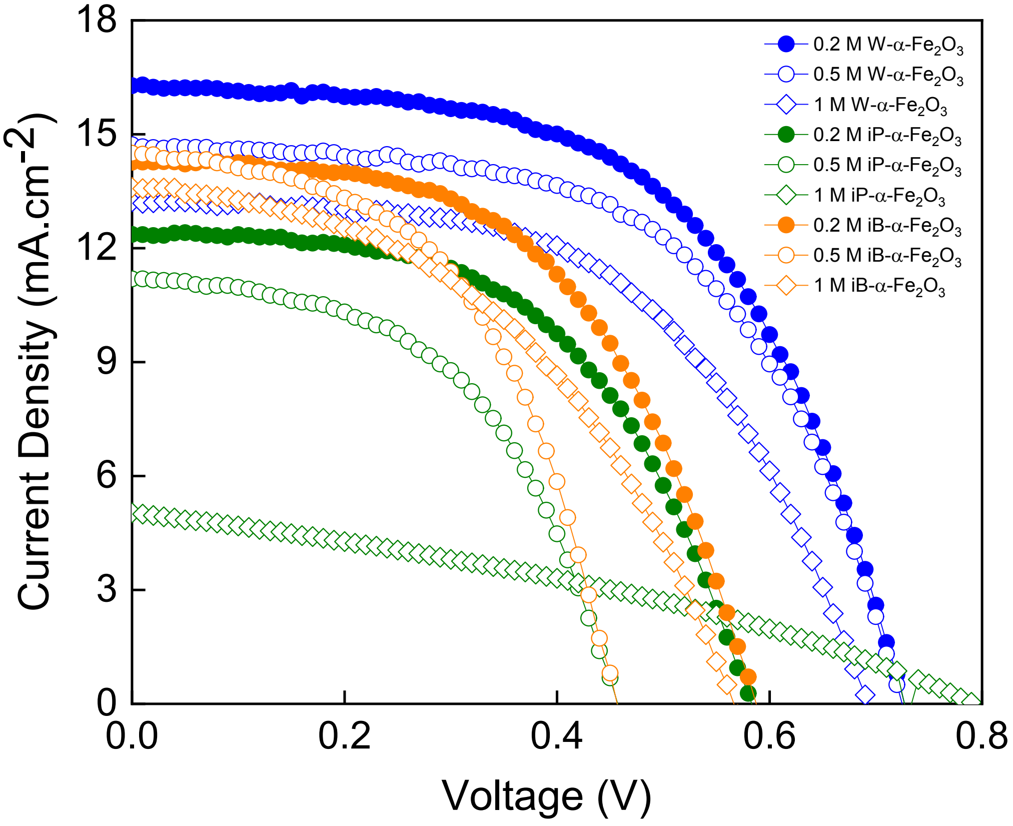

Figure S*6*. Light J-V curves of perovskite solar cells with α-Fe_2O_3 ETLs in different solvents and concentrations.

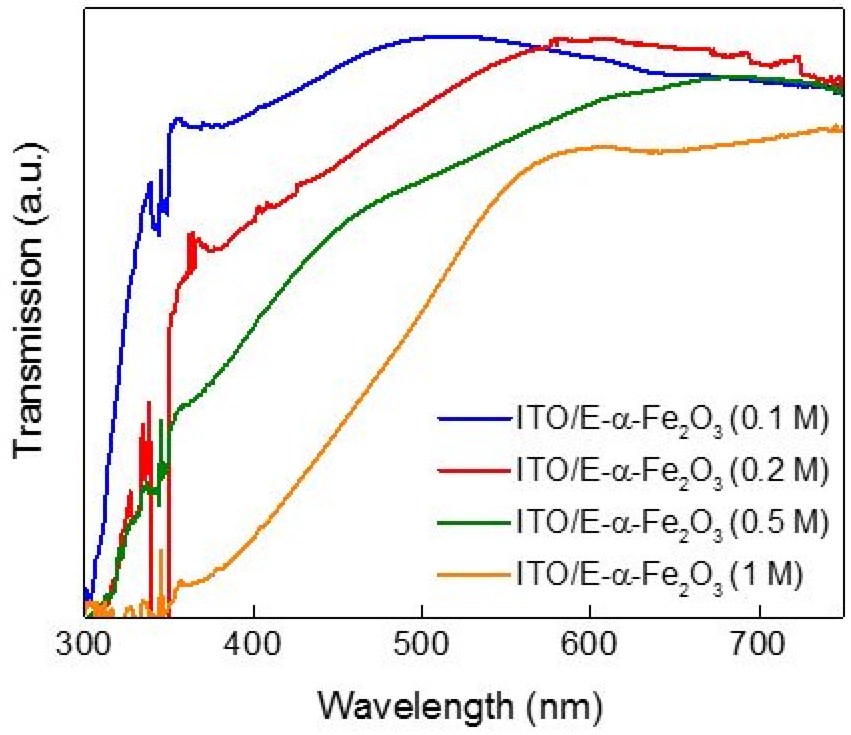

Figure S7. Transmission spectra of ITO/E-α-Fe_2O_3 thin films with different concentrations.

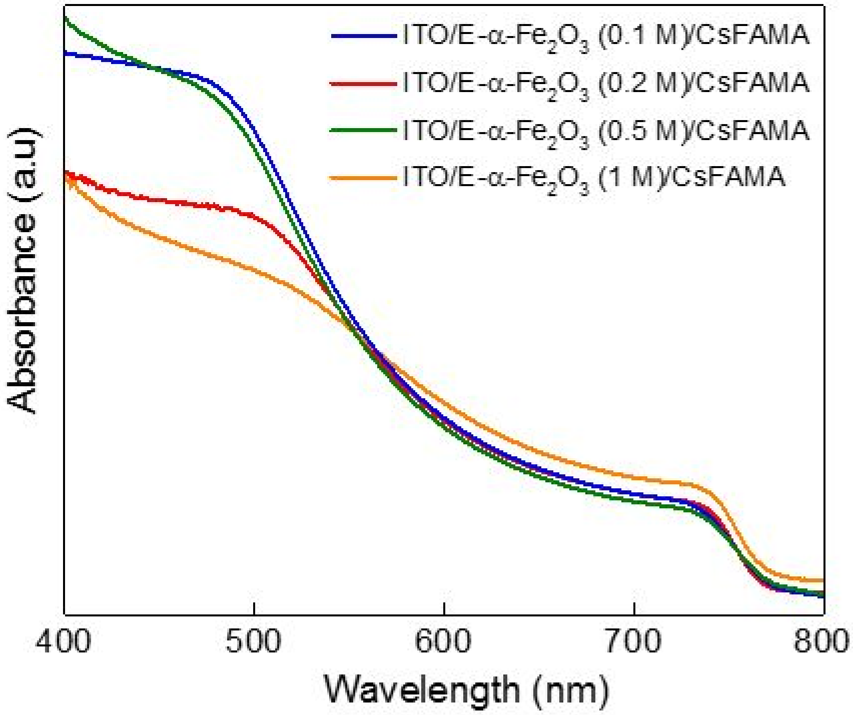

Figure S8. Absorption spectra of ITO/E-α-Fe_2O_3 thin films with different concentrations.

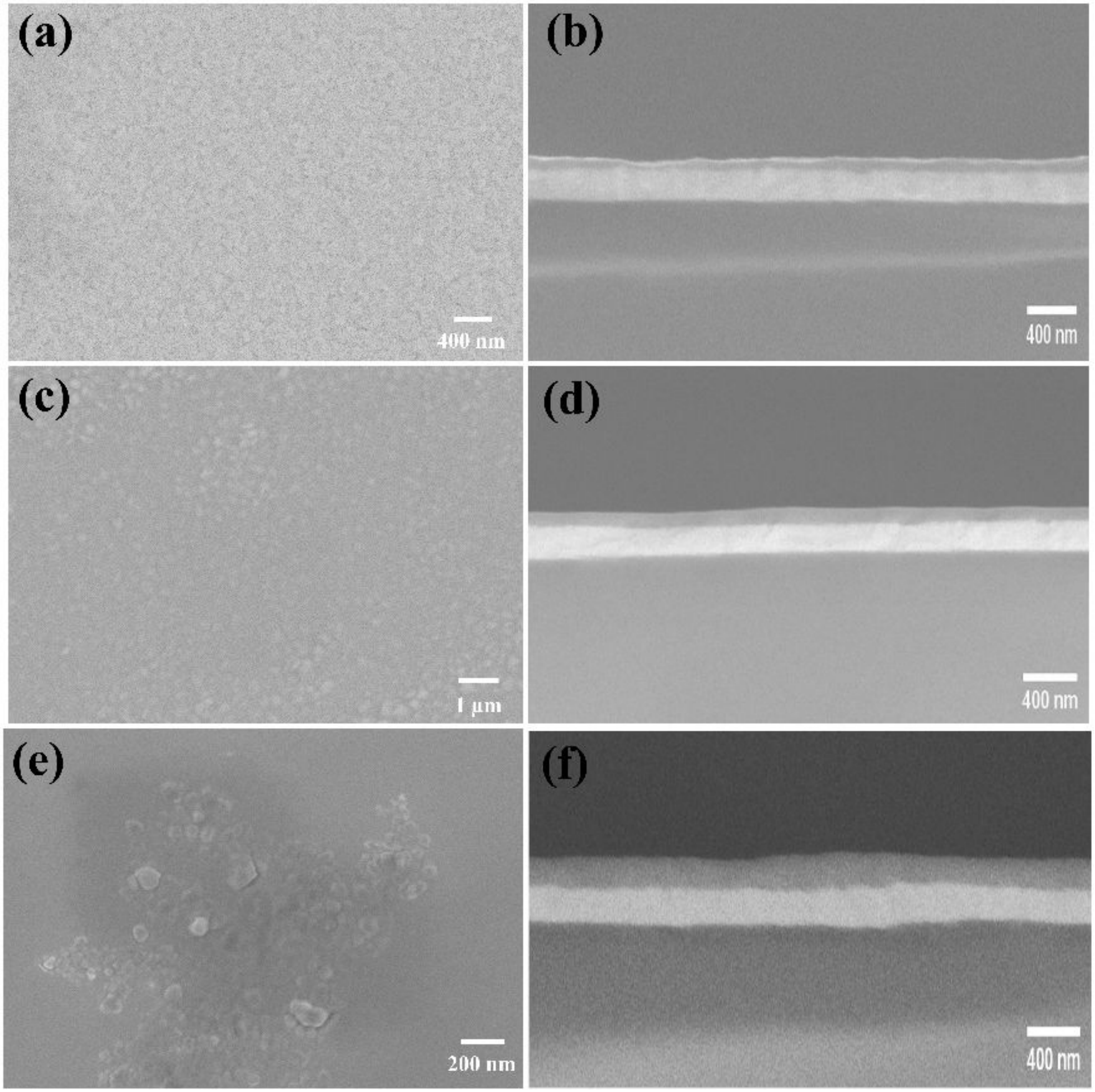

Figure S9. Top view SEM images of E-α-Fe_2O_3 compact layers deposited on ITO with concentrations: (a) 0.2 M, (c) 0.5 M, and (e) 1 M, Cross-sectional SEM images E-α-Fe_2O_3 compact layers deposited on ITO with concentrations: (b) 0.2 M, (d) 0.5 M, and (f) 1 M.

In order to confirm the reproducibility of our results, we fabricated at least 12 devices for each molar concentration and measured their photovoltaic performance (Figure S10). The statistical analysis showed a narrow distribution of the photovoltaic parameters as well as the highest performance for 0.2 M E-α-Fe_2O_3 ETL confirming the reproducibility and reliability of our results.

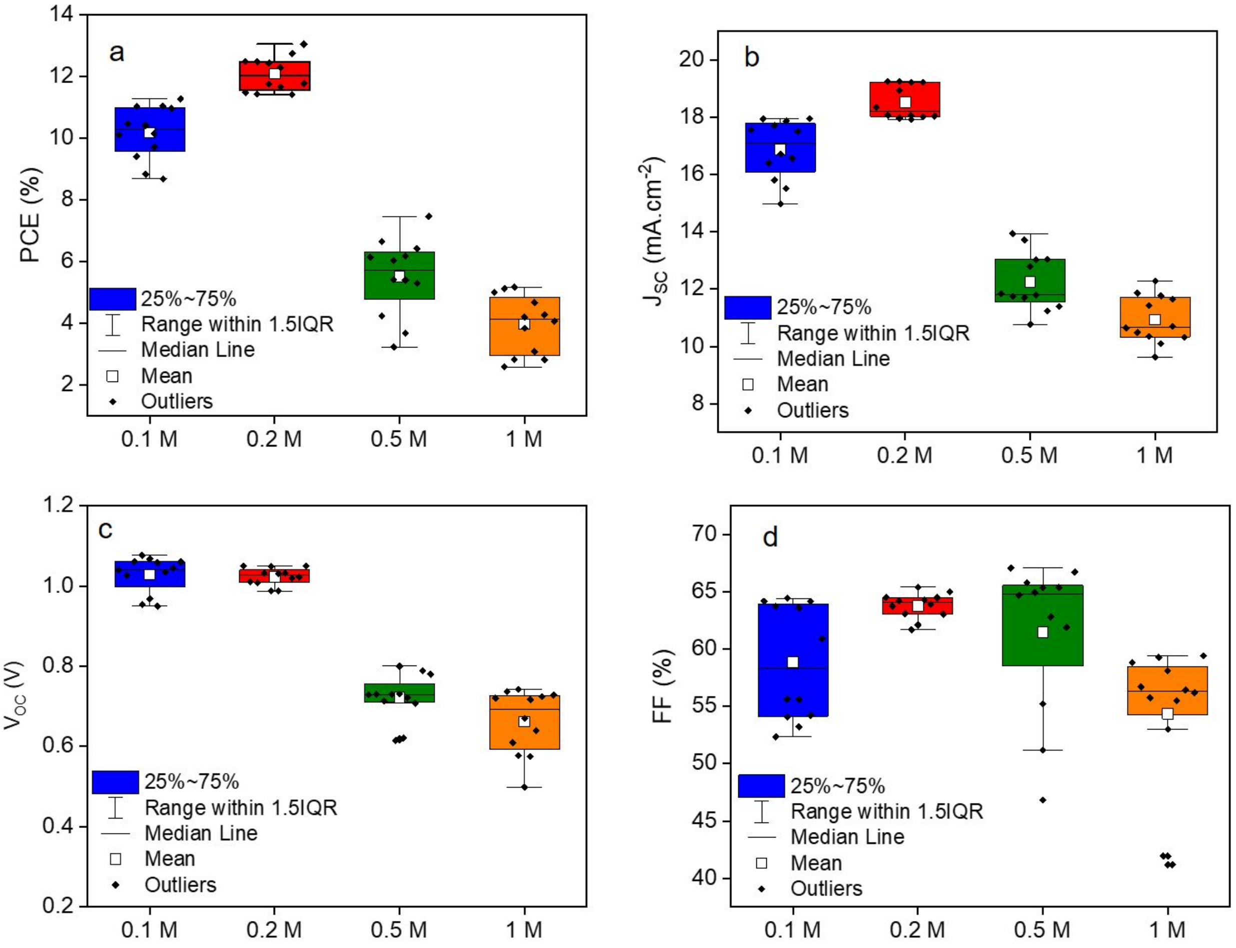

Figure S3. Statistical analysis of the photovoltaic parameters of the PSCs made using different molar concentrations of the α-Fe_2O_3 ETL in ethanol): (a) PCE, (b) J_{SC}, (c) V_{OC}, and (d) FF.

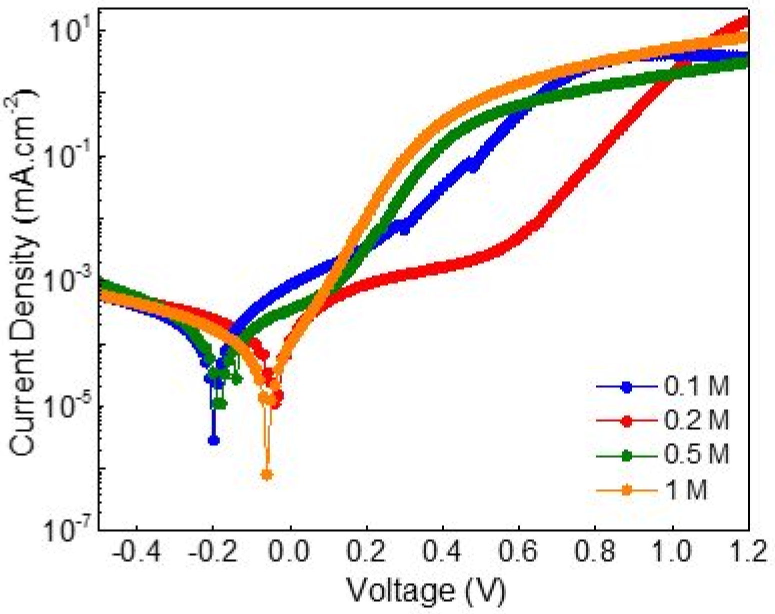

Figure S4. Dark J-V curves of perovskite solar cells with E-α-Fe_2O_3 ETL in different concentrations.

Figure S12a depicts the top view SEM image of CsFAMA perovskite deposited over-optimized 0.2 M E-α-Fe_2O_3 ETL. The dense and crack-free surface coverage of the perovskite film was observed for the 0.2 M E-α-Fe_2O_3 ETL. S12b and Figure S13 show the top view AFM image of CsFAMA perovskite deposited over-optimized 0.2 M E-α-Fe_2O_3 ETL. The large size grains are evident from AFM images with clearly visible grain boundaries. The longitudinal fusion of grain boundaries and vertical growth of the perovskite as in Figure S12c confirmed the better electronic extraction and transport from perovskite to ETL interface. Figure S12d depicts the cross-sectional SEM image of a 0.2 M E-α-Fe_2O_3 ETL-based perovskite solar cell. We further examined the morphology of perovskite for each concentration of E-α-Fe_2O_3 ETL (Figure S14). The crack-free dense morphology was observed for each ETL affirming the superiority and reproducibility of triple cation perovskite.

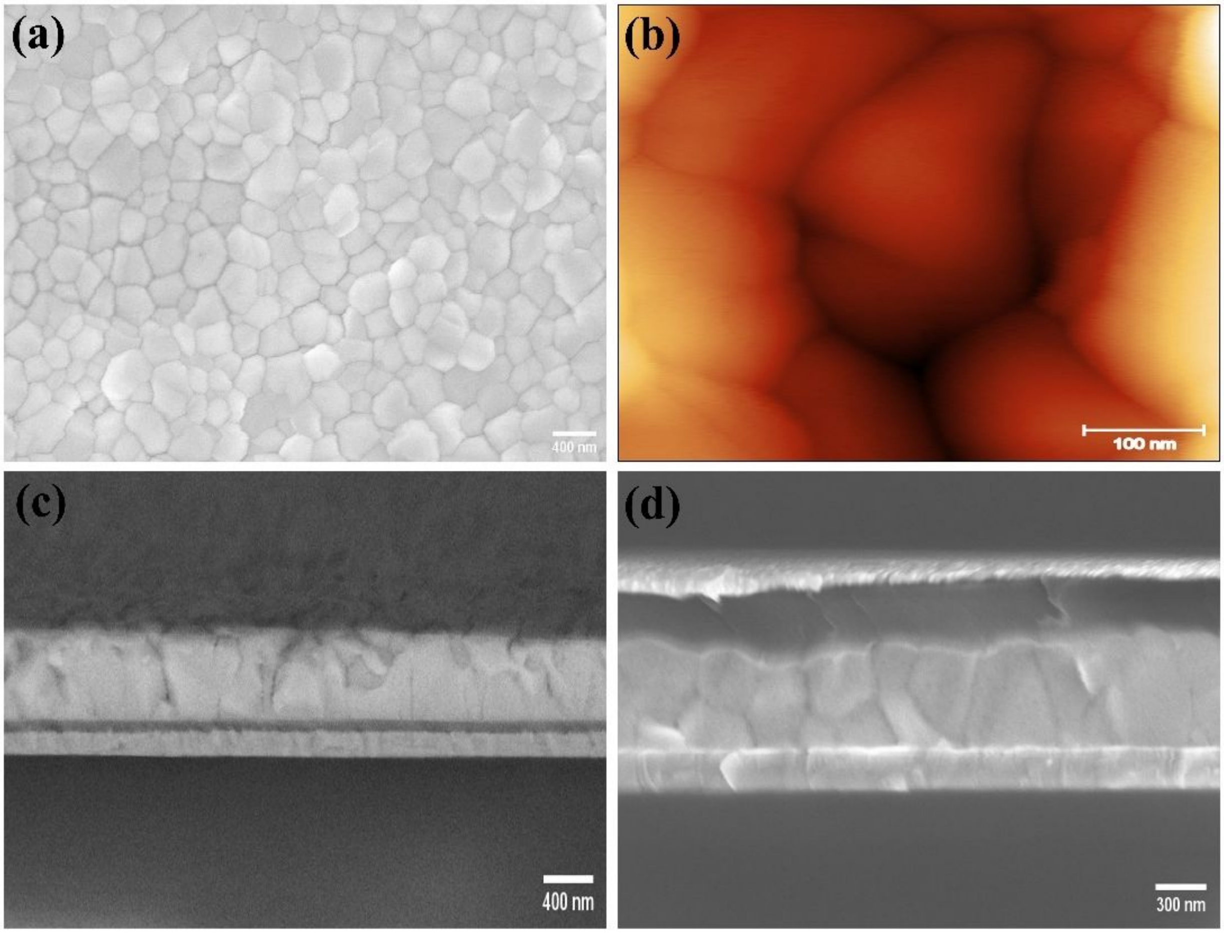

Figure S5. (a) Top view SEM image of CsFAMA perovskite thin films deposited on ITO/E-α-Fe_2O_3 ETL, (b) Top view AFM image of CsFAMA perovskite thin films deposited on ITO/E-α-Fe_2O_3 ETL, (c) Cross-sectional SEM image of CsFAMA perovskite thin films deposited on ITO/E-α-Fe_2O_3 ETL (0.5 M), and (d) cross-sectional SEM image of a full device with optimized solvent and optimized concentration

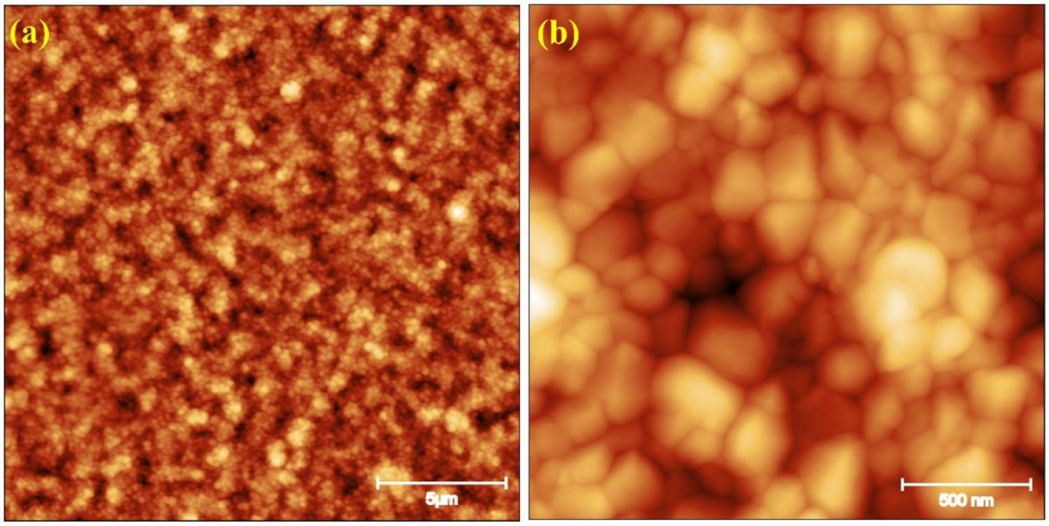

Figure S6. Top view AFM images of CsFAMA perovskite thin films deposited on ITO/E-α-Fe_2O_3 ETL in different resolutions, (a) 5 μm, and (b) 500 nm.

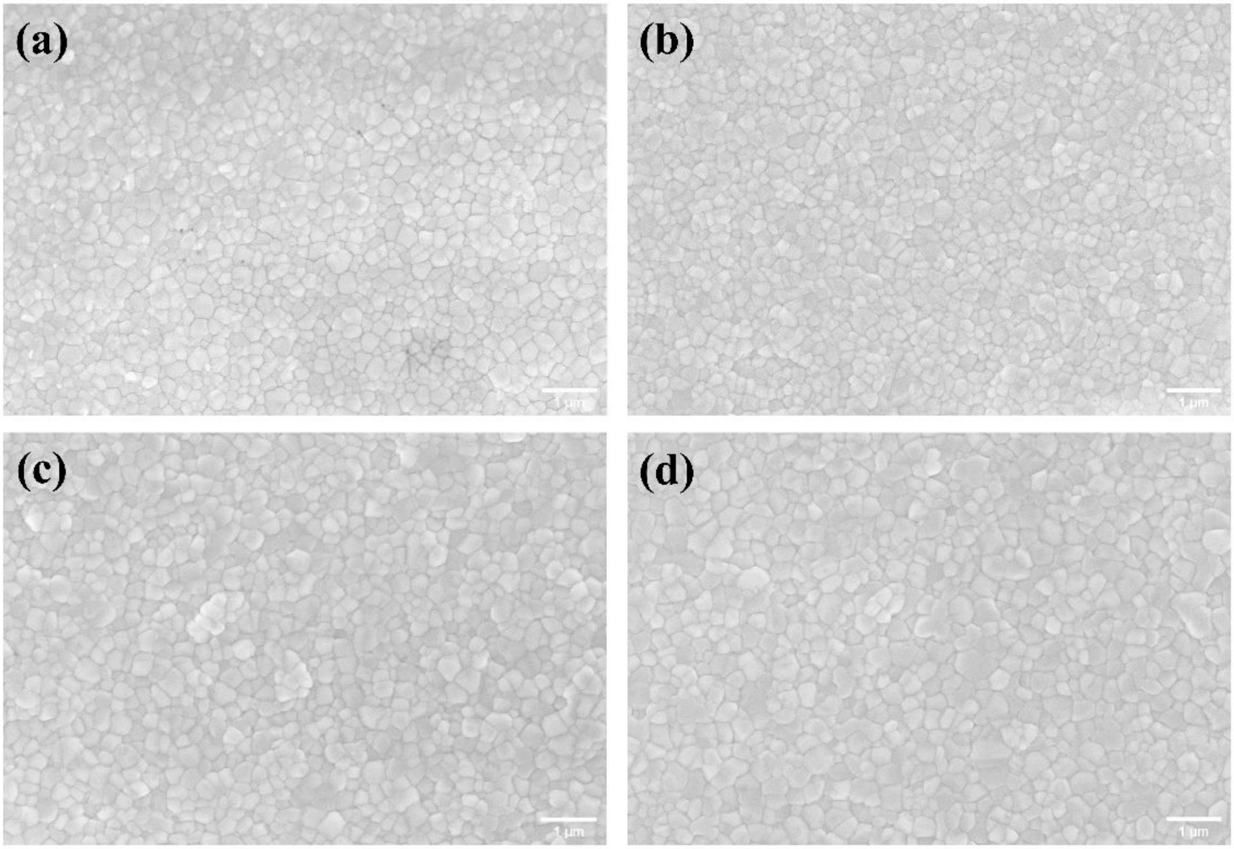

Figure S7. Top view SEM images of CsFAMA perovskite deposited on E-α-Fe_2O_3 ETLs in different concentrations, (a) 0.1 M, (b) 0.2 M, (c) 0.5 M, and (d) 1 M.

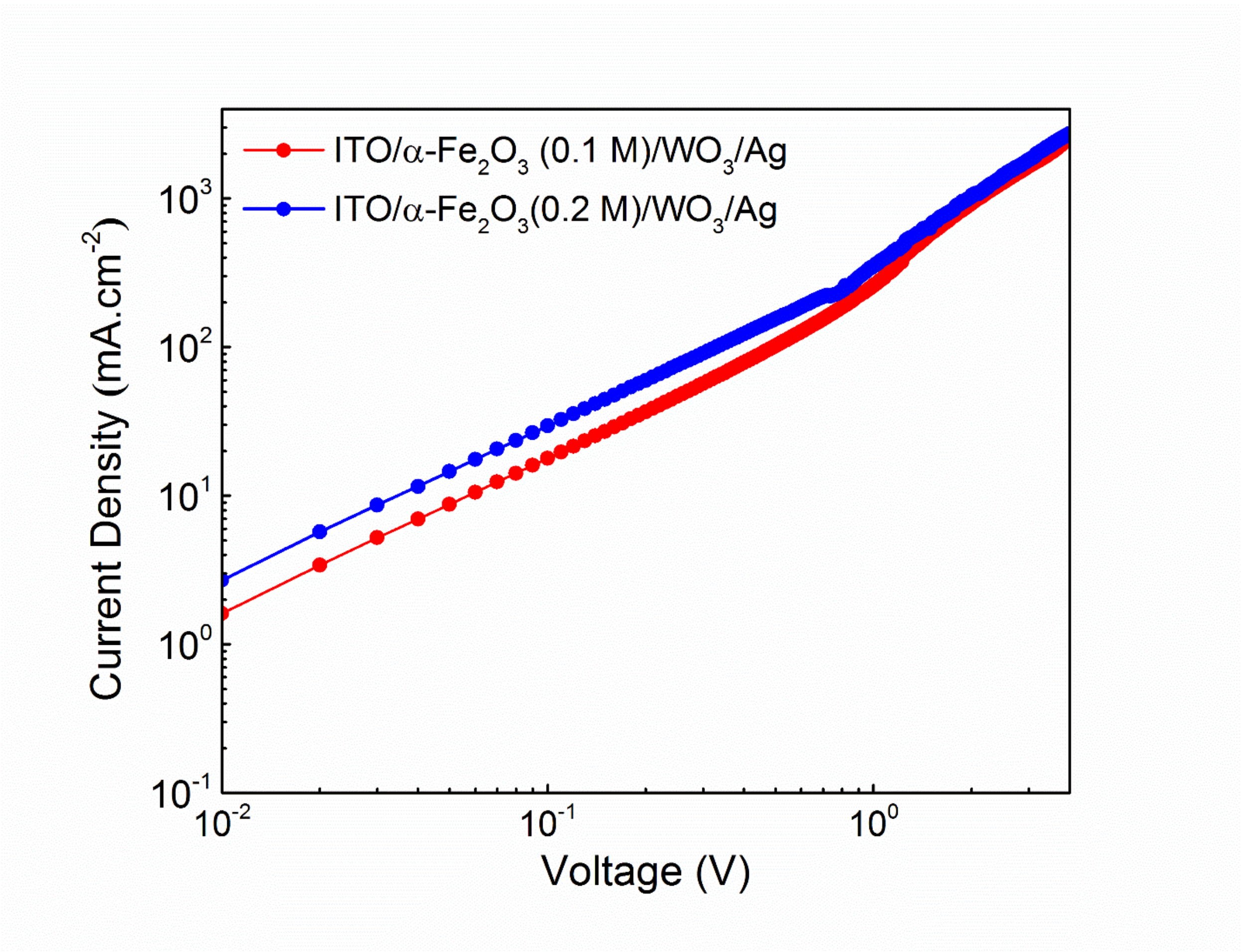

Figure S8. SCLC measurements of the electron-only device with 0.1 M and 0.2 M E-α-Fe_2O_3 ETLs in configuration (ITO/α-Fe_2O_3/WO_3/Ag).

Table S1 Fast and slow components for the PL decay and their corresponding proportions with ITO/CsFAMA

Description	τ_1 (ns)	A_1 (%)	τ_2 (ns)	A_2 (%)	τ_{ave} (ns)
**ITO/CsFAMA**	6.39	31.28	241.33	68.72	238.52

Table S2 Fast and slow components for the PL decay and their corresponding proportions with ITO/α-Fe_2O_3/CsFAMA in different solvents

Description	τ_1 (ns)	A_1 (%)	τ_2 (ns)	A_2 (%)	τ_{ave} (ns)
**W-α-Fe_2O_3**	3.40	69.29	22.81	30.71	17.93
**E-α-Fe_2O_3**	4.82	70.76	31.97	29.24	24.71
**iP-α-Fe_2O_3**	7.58	52.95	92.17	47.05	85.00
**iB-α-Fe_2O_3**	2.22	62.72	21.44	37.28	18.59

Table S3 Photovoltaic parameters of PSCs using α-Fe_2O_3 made from different solvents and different concentrations

ETL	Concentration	J_{SC} (mA.cm^{-2})	V_{OC} (V)	FF (%)	PCE (%)
W-α-Fe_2O_3	0.2 M	16.27	0.72	56.67	6.64
	0.5 M	14.73	0.72	57.63	6.11
	1 M	13.22	0.69	55.62	5.07
iP-α-Fe_2O_3	0.2 M	12.35	0.58	54.08	3.87
	0.5 M	11.19	0.45	51.67	2.60

	1 M	5.03	0.79	34.16	1.36
$iB-\alpha-Fe_2O_3$	0.2 M	14.26	0.58	54.25	4.49
	0.5 M	14.50	0.45	51.58	3.37
	1 M	13.60	0.56	46.14	3.51

Table S4 Fast and slow components for the PL decay and their corresponding proportions with ITO/E-α-Fe_2O_3/CsFAMA with different concentrations.

**Description**	**Concentration**	**τ_1 (ns)**	**A_1 (%)**	**τ_2 (ns)**	**A_2 (%)**	**τ_{ave} (ns)**
**ITO/E-α-Fe_2O_3/CsFAMA**	0.1 M	5.05	70.51	32.42	29.49	24.99
	0.2 M	2.55	90.87	18.99	9.13	9.59
	0.5 M	6.12	60.72	65.37	39.28	57.88
	1 M	2.87	55.17	109.29	44.83	105.96
